# Artemisinin-based combination therapy does not measurably reduce human infectiousness to vectors in a setting of intense malaria transmission

**DOI:** 10.1186/1475-2875-11-118

**Published:** 2012-04-18

**Authors:** Bernadette J Huho, Gerard F Killeen, Heather M Ferguson, Adriana Tami, Christian Lengeler, J Derek Charlwood, Aniset Kihonda, Japhet Kihonda, S Patrick Kachur, Thomas A Smith, Salim MK Abdulla

**Affiliations:** 1 Ifakara Health Institute, Dar-es-Salaam, Tanzania; 2 Swiss Tropical and Public Health Institute, Basel, Switzerland; 3 Liverpool School of Tropical Medicine, Liverpool, UK; 4 Division of Infection and Immunity, University of Glasgow, G12 8TA, Glasgow, UK; 5 Department of Medical Microbiology, University Medical Center Groningen, Groningen, The Netherlands; 6 Royal Tropical Institute, Biomedical Research, Amsterdam, The Netherlands; 7 Malaria Branch, US Centers for Disease Control and Prevention, Atlanta, USA; 8 University of Basel, Petersplatz 1, Basel CH-4003, Switzerland

**Keywords:** Malaria, Artemisinin-based combination therapy, Transmission reduction, Malaria

## Abstract

**Background:**

Artemisinin-based combination therapy (ACT) for treating malaria has activity against immature gametocytes. In theory, this property may complement the effect of terminating otherwise lengthy malaria infections and reducing the parasite reservoir in the human population that can infect vector mosquitoes. However, this has never been verified at a population level in a setting with intense transmission, where chronically infectious asymptomatic carriers are common and cured patients are rapidly and repeatedly re-infected.

**Methods:**

From 2001 to 2004, malaria vector densities were monitored using light traps in three Tanzanian districts. Mosquitoes were dissected to determine parous and oocyst rates. *Plasmodium falciparum* sporozoite rates were determined by ELISA. Sulphadoxine-pyrimethamine (SP) monotherapy was used for treatment of uncomplicated malaria in the contiguous districts of Kilombero and Ulanga throughout this period. In Rufiji district, the standard drug was changed to artesunate co-administered with SP (AS + SP) in March 2003. The effects of this change in case management on malaria parasite infection in the vectors were analysed.

**Results:**

*Plasmodium falciparum* entomological inoculation rates exceeded 300 infective bites per person per year at both sites over the whole period. The introduction of AS + SP in Rufiji was associated with increased oocyst prevalence (OR [95%CI] = 3.9 [2.9-5.3], p < 0.001), but had no consistent effect on sporozoite prevalence (OR [95%CI] = 0.9 [0.7-1.2], p = 0.5). The estimated infectiousness of the human population in Rufiji was very low prior to the change in drug policy. Emergence rates and parous rates of the vectors varied substantially throughout the study period, which affected estimates of infectiousness. The latter consequently cannot be explained by the change in drug policy.

**Conclusions:**

In high perennial transmission settings, only a small proportion of infections in humans are symptomatic or treated, so case management with ACT may have little impact on overall infectiousness of the human population. Variations in infection levels in vectors largely depend on the age distribution of the mosquito population. Benefits of ACT in suppressing transmission are more likely to be evident where transmission is already low or effective vector control is widely implemented.

## Background

Currently, artemisinin-based combination therapy (ACT) is used as first-line treatment of uncomplicated malaria in most countries in sub-Saharan Africa. In addition to killing the asexual blood stages that cause disease and, therefore, terminating otherwise lengthy, persistently transmissible infections [1-3], artemisinins are gametocytocidal, killing the immature sexual stages of malaria parasites eventually responsible for infecting mosquitoes [4,5]. While non-gametocyctocidal drugs will also cure otherwise lengthy infections and reduce the period of infectiousness to mosquitoes, gametocytes will remain in the cured individual for some time, allowing for transmission.

In principle, through their combined impacts upon both the short-term infectiousness of treated individuals, and perhaps more importantly [6], upon the long-term duration of infection and therefore infectiousness, ACT might reduce the reservoir of parasites in the human population that eventually infects mosquitoes.

The provision of ACT for treatment of uncomplicated malaria has been associated with reduced malaria incidence in diverse settings with modest transmission intensity [4,7,8]. This implies that ACT may effectively reduce human-to-mosquito and consequently mosquito-to-human transmission under normal conditions of programmatic use, as has been suggested in individually randomized, controlled trials evaluating the infectiousness of patients receiving ACT [9-11].

Determination of the proportion of humans harbouring gametocytes following ACT treatment may not accurately estimate human population infectiousness since infectiousness seems only loosely correlated to gametocyte density [12,13]. In malaria-endemic settings, humans can be infectious to mosquitoes even in the absence of patent gametocytaemia, regardless of treatment [1,14-16]. While human-to-mosquito feeding experiments with laboratory-reared mosquitoes are very useful, they do not capture parasite infection and selection dynamics in the context of their human host populations [17-20] and are not necessarily representative of the wild mosquito populations which have natural feeding biases influenced by host age and infection status [21-24]. Estimation of the human infectious reservoir therefore requires analysis of the infection status of wild-caught mosquitoes.

A pre-post observational study with a contemporaneous comparison group was used to evaluate the impact of case management with ACT delivered through fixed health facilities in two sites in rural Tanzania with intense malaria transmission [25]. Both the intervention and comparison sites used sulphadoxine-pyrimethamine (SP) as first-line treatment of malaria in 2001–2003. In March 2003, the ACT, artesunate co-administered with SP (AS + SP), was introduced as a first-line treatment of malaria in the intervention site while SP continued to be used for first-line treatment in the comparison site. To assess the impact of ACT introduction on malaria transmission, concurrent measures of oocyst and sporozoite prevalence in the mosquito-vector population in both the intervention and comparison districts, before and after the introduction of AS + SP, were carried out and used to directly determine the infectiousness of the human population to mosquitoes, and of mosquitoes to humans.

## Methods

### Study site

**Figure 1:**
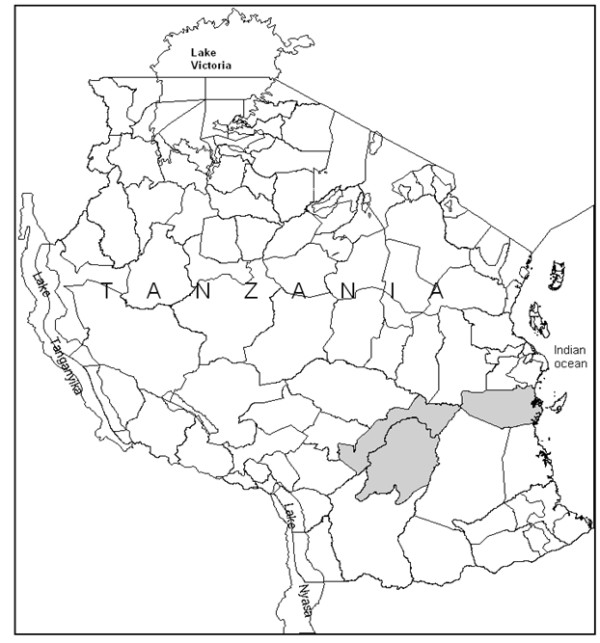
**Map of the study districts**. A: Rufiji; B: Kilombero; C: Ulanga.

This study was conducted in two rural sites in southeastern Tanzania. Rufiji District, the intervention site, is located at the mouth of the Rufiji River, extends across latitudes 7° 47′ and 8° 03′S and longitudes 38° 62′ and 39° 17′E with a population of about 202,001 inhabitants [26,27]. Kilombero and Ulanga Districts, the comparison site, form the valley of the Kilombero River, one of the main tributaries of the Rufiji and are situated between latitudes 8°00'–8°35'S, longitudes 35°58'–36°48'E and have a combined population of 514,891 inhabitants [27,28] (Figure 1). Both Rufiji and Kilombero-Ulanga Districts have achieved relatively high coverage of largely untreated bed nets [29,30] and are characterized by a hot climate with an erratic rainy season from November to May. In Rufiji, the average annual precipitation is 800–1,000 mm while Kilombero-Ulanga receives 1,200-1,800 mm. In both settings, malaria caused largely by *Plasmodium falciparum *[31] is one of the biggest health problems perceived by the local community and reported by the health services [32]. It is primarily transmitted by *Anopheles gambiaeAnopheles arabiensis* and *Anopheles funestus*. Transmission is intense and perennial despite marked seasonality in mosquito densities, which peak with the rains [31,33].

### Study design

The detailed description of the study is reported elsewhere [25]. Briefly, a pre-post observational study with a non-randomized comparison site was conducted. Both sites used SP monotherapy as a first-line anti-malarial delivered through health facilities from 2001. In March 2003, the Council Health Management Team implemented AS + SP combination therapy as the first-line anti-malarial provided free of charge through all the fixed health facilities in Rufiji District, the intervention site. SP monotherapy continued to be the first-line anti-malarial in Kilombero-Ulanga, the comparison site, as well as in the rest of the country. Here, SP was available free of charge only to pregnant women and children under five years of age.

### Mosquito data collection

In each site, anopheline indoor biting rates were determined by overnight trapping of host-seeking mosquitoes using Centers for Disease Control and Prevention (CDC) light traps. The two sites used slightly different household selection procedures. Sampling in Kilombero-Ulanga occurred from October 2001 to August 2004 and was based on repeated sampling every six months of 25 clusters of households selected by stratified random sampling, using the sub-village (*kitongoji*) as the first level and index household as the second level of randomization [29]. Trapping was carried out on 538 different nights, with an average of 4.9 traps per night. The traps were clustered in houses around the index house, but out of sight of each other.

In Rufiji, the period sampled included a 17-month pre-intervention period of October 2001 to February 2003 and a 19-month post-intervention period of March 2003 to September 2004. Individual households were randomly selected monthly from the same demographic surveillance sample frame used for surveys of human malaria infection [25]. Trapping was carried out on 850 distinct dates, with an average of 6.6 traps per night.

Light traps were installed about 0.5 m above the floor, next to the foot of the bed of the selected person who slept under a mosquito net. No attempt was made to differentiate between treated and untreated nets in the field as this proved impractical during routine field surveys and insecticide treatment has only a minor effect on sampling efficiency [29,34]. On occasions when the selected individual for light trap sampling lacked a net, he or she was provided with an untreated net for the nights during which they participated.

Once collected, mosquitoes were counted and sorted by species in the field. Where this was feasible, blood-fed female *An. gambiae* s.l. and *An. funestus* were held in a cup and fed on sugar water until the blood meal was digested, this period ranges from two to three days depending on temperature. Then, the mid-guts of these mosquitoes were dissected in normal saline and stained with 2% mercurochrome for examination of oocysts by light microscopy [35]. The remaining parts of the dissected mosquitoes as well as other undissected anophelines were routinely stored in Eppendorf tubes with a small quantity of silica gel. Mosquitoes were subsequently independently tested for circumsporozoite protein (CSP) by ELISA [36] in a central laboratory at Ifakara Health Institute. At each site, a different technician conducted the mosquito dissections and examinations for the presence of oocysts. Laboratory technicians performing the CSP ELISA were blinded to the oocyst status and source of the mosquitoes to avoid possible biases in the determination of sporozoite infection status.

### Ethical approval

Ethical approval was obtained from the Medical Research Coordination Committee of the National Medical Research Coordination Committee of National Institute for Medical Research, Tanzania (Reference number NIMR/HQ/R.8a/VOL.VIII, dated April 2000).

### Data analysis

The overall objective of the analysis was to determine the relationship between the introduction of ACT and the infectiousness of the human population, as reflected by infection prevalence in local vector populations. The outcome measures reflecting human-to-mosquito transmission were the infection status of individual mosquitoes, with the primary and secondary effects defined by the presence of oocysts or sporozoites, respectively, within the two study zones. The proportions of mosquitoes with oocysts and sporozoites (the oocyst and sporozoite rates, respectively) were estimated independently for groups of mosquitoes collected before and after the introduction of ACT in the intervention site. Multivariate logistic regression models with terms for study site (intervention versus comparison), period of mosquito collection (pre-intervention versus post-intervention), intervention (availability of ACT versus SP monotherapy), and species of mosquito (*An. gambiae* s.l. versus *An. funestus*), were used to assess the impact of the introduction of ACT on oocyst and sporozoite prevalence. Statistical significance was defined as a p-value ≤0.05. All statistical analyses were executed using SPSS 15.0 (SPSS Inc, Chicago, USA).

To measure mosquito-to-human malaria transmission intensity, the entomological inoculation rate (EIR) was calculated by multiplying the arithmetic mean mosquito-biting rate per night by the mean sporozoite prevalence for that vector species. EIR was calculated separately for the pre- and post- intervention periods. The biting rate for each mosquito species was obtained by dividing the mean catch of females in CDC light traps by published estimates from the Kilombero Valley of the relative sensitivity of CDC light traps relative to human landing catches of 0.30 and 0.68 for *An. gambiae* s.l. and *An. funestus*, respectively [37].

Infectiousness of humans to mosquitoes depends on *K*, the proportion of mosquitoes that are infected at any given feed. This cannot be measured directly, because infected mosquitoes may have received their infections either at the latest, or at a previous feed. There are various algorithms for estimating *K* from field-caught mosquitoes. All of these require both a measure of the proportions of mosquitoes that are infected, and a measure of the age distribution of the vectors. For the present study, *K* was estimated from the proportions of host-seeking mosquitoes with oocysts and the proportion that were parous using the following equation [38,39]:

(1) $$K_{O}=\frac{1-\frac{1}{M}}{1-\frac{M}{R}}$$

Where: *M* is the proportion of parous mosquitoes among those dissected and *R* is the proportion of dissected mosquitoes with oocysts (the immediate oocyst rate). The standard error of *K*_*O*_ was determined as described previously [38].

## Results

**Figure 2:**
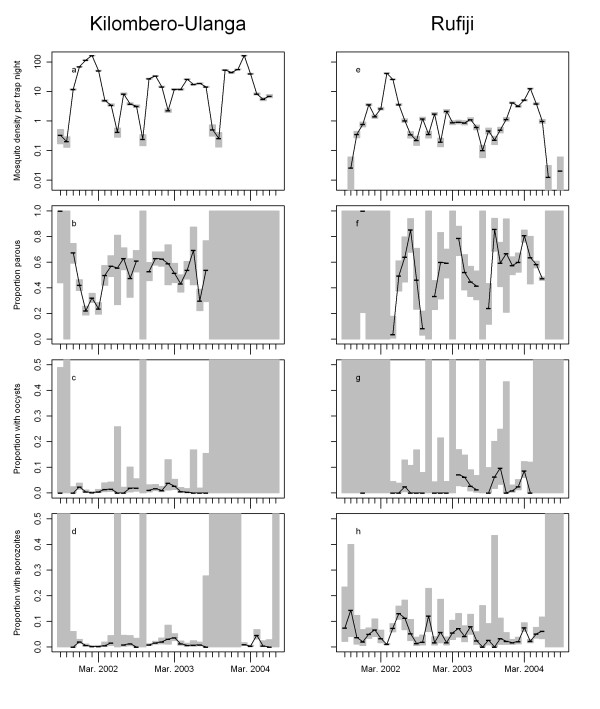
***Anopheles gambiae*s.l. density (panels a and e), proportion parous (panels b and f), proportion with oocysts (panels c and f) and proportion with sporozoites (panels d and g) for Kilombero-Ulanga (panels a – d) and Rufiji (panels e – g) districts by month.** Horizontal black lines represent observed values, grey bars represent 95% confidence intervals. Subsequent non-missing values are connected by thin black lines.

**Figure 3:**
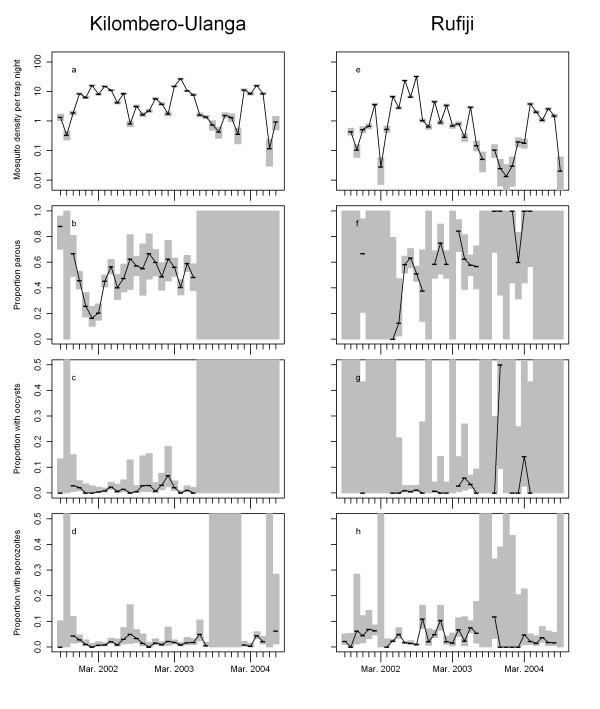
***Anopheles funestus*density (panels a and e), proportion parous (panels b and f), proportion with oocysts (panels c and f) and proportion with sporozoites (panels d and g) for Kilombero-Ulanga (panels a – d) and Rufiji (panels e – g) districts by month**. Horizontal black lines represent observed values, grey bars represent 95% confidence intervals. Subsequent non-missing values are connected by thin black lines.

**Figure 4:**
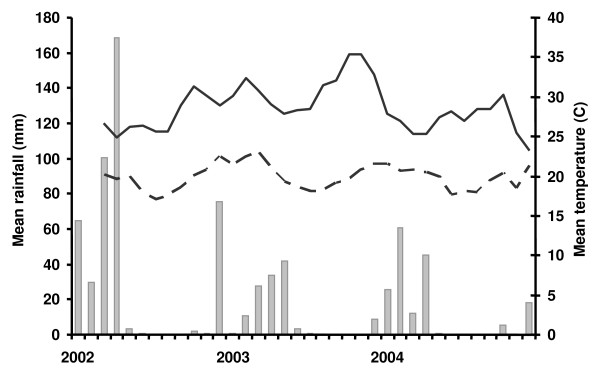
**Temperature and rainfall for Rufiji for the period of 2002–2004.** The bars represent the rainfall per month (left axis), the lines show the monthly maximum (solid line) and minimum temperature (broken line), right axis. Values are based on remote sensing. Rainfall data were obtained from the Africa Data Dissemination Service (*ADDS*) [53] and temperature data from the National Aeronautics and Space Administration [54].

In Rufiji, 11,883 *An. gambiae* s.l. and 13,434 *An. funestus* were sampled before ACT introduction, while 5,826 *An. gambiae* s.l. and 2,626 *An. funestus* were sampled after ACT introduction. In the comparison site: Kilombero-Ulanga, 50,694 *An. gambiae* s.l. and 9,615 *An. funestus* were sampled before and 27,559 *An. gambiae* s.l. and 8,381 *An. funestus* after ACT introduction in Rufiji. The density of anophelines as well as the parous rate varied seasonally and strongly between years (Figures 2 and 3). Fewer mosquitoes were caught post the intervention in Rufiji, but both 2003 and 2004 were very dry years (Figure 4) and this was presumably the main factor affecting mosquito densities.

**Figure 5:**
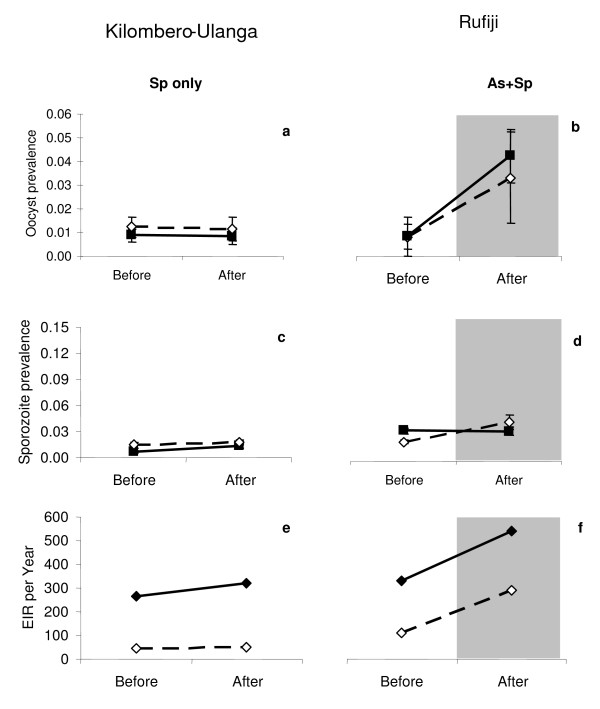
**Trends in mosquito infection prevalence.** Observed trends of mosquito oocyst (**a** &**b**) and sporozoite (**c** &**d**) prevalence before and after the onset of an artesunate-based effectiveness trial, error bars correspond to 95% confidence interval. A comparison can be made for *Anopheles gambiae* s.l. (straight line and dark squares) and *Anopheles funestus* (dotted line, white squares). Panels E & F show the trends in malaria transmission. The shading on the graphs serves to indicate the transition from before and after the addition of AS to SP.

Oocyst prevalence in Rufiji increased substantially between the pre-intervention and post-intervention period (χ^2^ = 11.9, p <0.001 for *An. gambiae*, χ^2^ = 11.1, p <0.001 for *An. funestus*) with an odds ratio (estimated from a multivariable logistic regression, allowing for site, species, and time period) of 3.9 [95%CI: 2.9-5.3] (Figure 5). However, the confidence intervals for both *An. funestus* and *An. gambiae* s.l. oocyst rates were wide (Figures 2 and 3 respectively) because of the considerable inter-month variation. No significant changes (χ^2^ = 0.01, p =0.9 for *An. gambiae*, χ^2^ = 0.04, p = 0.8 for *An. funestus*) were observed in Kilombero-Ulanga (Table 1, Figures 2, 3 and 4). Sporozoite prevalence also increased significantly in Rufiji for *An. funestus* (χ^2^ = 37.3, p < 0.001), but not for *An*. *gambiae* s.l. (χ^2^ = 0.02, p =0.9) so overall there was little effect (OR [95%CI] = 0.9[0.7-1.2], p = 0.51) (Table 2) while in Kilombero-Ulanga the sporozoite prevalence increased significantly for *An*. *gambiae* s.l. (χ^2^ = 21.6, p <0.001), but not for *An. funestus* (χ^2^ = 1.7, p =0.19). These formal statistical comparisons between pre- and post-intervention periods must be viewed cautiously in the context of the considerable seasonal and inter-annual variation in both mosquito densities, and in the numbers of mosquitoes that were analysed for each outcome. The age distribution of the mosquito populations, as indicated by the parous rates, also varied considerably over time, reflecting variations in both mosquito survival and recruitment rate to the vector populations. Environmental variation (Figure 4) is probably the main determinant of longitudinal patterns in mosquito bionomics. Because of the profound inter-annual differences we did not attempt to adjust these analyses for seasonality.

**Table 1:** Prevalence of mosquitoes infected with oocysts and sporozoites and entomological inoculation rate (EIR) in Rufiji and Kilombero-Ulanga Districts by Anopheline species and time period

	**Time period (Anti-malarial in use)**	**Oocyst prevalence**		**Sporozoite prevalence**		**EIR**
		**n/N**	**% [95% CI]**	**n/N**	**% [95% CI]**	
**Rufiji**						
*An. funestus*	January 2002-February 2003 (SP)	9/1094	0.82 [0.29-1.36]	321/14861	0.022 [0.019-0.024]	108
	March 2003- September 2004 (AS + SP)	11/330	3.33 [1.40-5.27]	99/2273	0.044 [0.035-0.052]	288
*An. gambiae s.l*	January 2002-February 2003 (SP)	4/475	0.84 [0.02-1.66]	291/8665	0.034 [0.030-0.037]	332
	March 2003-September 2004 (AS + SP)	51/1195	4.27 [3.12-5.41]	215/6475	0.033 [0.029-0.038]	538
**Kilombero-Ulanga**						
*An. funestus*	January 2002-February 2003 (SP)	31/2518	1.23 [0.80-1.66]	63/4353	0.014 [0.011-0.018]	45
	March 2003- August 2004 (SP)	21/1806	1.16 [0.67-1.66]	117/6576	0.018 [0.015-0.021]	50
*An. gambiae s.l*	January 2002-February 2003 (SP)	40/4506	0.89 [0.61-1.16]	63/9333	0.007 [0.005-0.008]	267
	March 2003- August 2004 (SP)	24/2765	0.87 [0.52-1.21]	128/9372	0.014 [0.011-0.016]	320

EIR = Entomological inoculation rate expressed as infectious mosquito bites per person per year.

95% CI = 95% confidence interval.

SP = Sulphadoxine-pyrimethamine.

AS + SP = Artesunate co-administered with sulphadoxine-pyrimethamine.

**Table 2:** Factors associated oocyst and sporozoite prevalence in Anopheline vectors in Rufiji and Kilombero-Ulanga Districts, January 2002-July 2004

**Variable**	**Oocyst prevalence**		**Sporozoite prevalence**	
	**OR [95% CI]**	**P value**	**OR [95% CI]**	**P value**
**District**				
Kilombero-Ulanga	Referent	Referent	Referent	Referent
Rufiji	0.72 [0.38-1.37]	0.31	2.51 [2.22-2.84]	<0.001
**Period**				
January 2002-February 2003	Referent	Referent	Referent	Referent
March 2003-July 2004	1.09 [0.76-1.58]	0.63	1.44 [1.28-1.61]	<0.001
**Anti-malarial in use**				
SP	Referent	Referent	Referent	Referent
AS + SP	3.91 [2.88-5.33]	<0.001	0.92 [0.72-1.18]	0.51
**Anopheline species**				
* An. gambiae s.l*	Referent	Referent	Referent	Referent
* An. funestus*	1.19 [0.88-1.61]	0.26	0.96 [0.85-1.07]	0.45

95% CI = 95% confidence interval.

SP = Sulphadoxine-pyrimethamine.

AS + SP = Artesunate co-administered with sulphadoxine-pyrimethamine.

Mosquito-to-human transmission, as estimated by the EIR, consistently exceeded 300 infective bites per person per year in both sites throughout the study period (Table 1). During both the pre-intervention and post-intervention time periods, the intervention site had the highest proportion of sporozoite-positive mosquitoes and, therefore, the highest EIR. The estimated EIR for both *An. gambiae* s.l. and *An. funestus* in the intervention site was higher after ACT introduction than before. This coincided with a possible increase in human-to-mosquito transmission implied by the observed increase in oocyst prevalence. In the absence of an increase in prevalence of sporozoites in *An. gambiae* s.l. it is impossible to draw any firm conclusions about effects on the infectious reservoir, which does not necessarily follow the EIR in endemic settings [39]. One clear conclusion though is that the introduction of ACT was not followed by signs of a decline in human-mosquito transmission.

The estimates of infectiousness of the human population were summarized at the level of the time period (pre- or post-policy change), by site, and by vector species (Table 3). The values of *K*_*O*_ were similar for both vector species, both sites and both time periods, with the exception of the pre-intervention values for Rufiji, which were very low. Much of the variation in sporozoite and oocyst prevalence can thus be attributed to variations in mosquito survival, which are accounted for by the term for the parous rate (*M*) in the formula for *K*_*O*_.

**Table 3:** **Oocyst-based estimates of human infectiousness (*Ko)***

	**Time Period (Anti-malarial in use)**	***Ko***	**[95%CI]**
**Rufiji**			
*An funestus*	January 2002-February 2003 (SP)	0.011	[0.000 - 0.016]
	March 2003-September 2004 (AS + SP)	0.029	[0.013 - 0.054]
*An gambiae* s.l.	January 2002-February 2003 (SP)	0.006	[−0.009 - 0.013]
	March 2003-September 2004 (AS + SP)	0.042	[0.028 - 0.057]
**Kilombero-Ulanga**			
*An funestus*	January 2002-February 2003 (SP)	0.034	[−0.001 - 0.025]
	March 2003-August 2004 (SP)	0.019	[−0.001 - 0.021]
*An gambiae* s.l.	January 2002-February 2003 (SP)	0.032	[0.000 - 0.020]
	March 2003-August 2004 (SP)	0.024	[−0.005 - 0.025]

The values estimated for *K* in the literature are extremely variable [39] but few of them are as low as the values measured pre-intervention in Rufiji. The values for Kilombero-Ulanga do not show any indication of a trend over time, and are higher than the pre-intervention Rufiji ones, suggesting that the low values cannot be attributed to the use of SP as treatment. There is no indication that the post-policy change values for Rufiji reduced *K* below the Kilombero value.

## Discussion

Despite numerous clinical studies demonstrating high cure rates and gametocytocidal effect of artemisinin derivatives [9,10,40,41], there is no evidence that this translates into any measurable impact on malaria transmission intensity at the population level in these Tanzanian sites. Although, the potential to reduce malaria transmission is widely cited, some mathematical models predict only a modest incremental impact of the routine use of ACT over non-gametocytocidal drugs in high transmission settings [19]. This observational study of the impact of routine delivery of ACT via health facilities provides some empirical support for this. Although, the parasitological study found a significant reduction in asexual parasitaemia prevalence following ACT introduction, this reduction was very modest (five percentage-points) and was not reflected in a measurable reduction of gametocytaemia prevalence in the human population [25] . In the present study, the most direct indicator of human-to-mosquito transmission, namely oocyst prevalence, was substantially higher after ACT introduction. It is unclear what caused this increase, particularly since the sporozoite prevalence did not increase at the same time (Table 2), only factors, in particular weather patterns (Figure 4) changed considerably between the two periods. Because environmental conditions and availability of mosquitoes for analysis varied erratically throughout the study period, it is not possible to formally separate inter-annual and seasonal variation from effects of the policy change, but the overall conclusion is that any ACT-related reductions in human-to-mosquito or mosquito-to-human transmission in the mosquito population were small.

Overall, these two large-scale, complementary studies of malaria parasite prevalence in both humans and mosquitoes did not detect any epidemiologically meaningful suppression of human population infectiousness following ACT introduction. However, mosquito population dynamics in Rufiji were clearly profoundly affected by variations in rainfall during the study period. Rainfall affects both the emergence rates of vectors, and probably (via effects on humidity) the survival of adult mosquitoes. This does not directly affect the infectiousness of the human population to mosquitoes, but has profound effects on malaria transmission as measured either by the EIR or the oocyst prevalence. The large variations in emergence rates and survival of mosquitoes very likely account for most of the variation in oocyst prevalence, though this cannot explain why infectiousness was so low during the first half of the study (prior to ACT) in Rufiji, or why the oocyst prevalence increased after ACT introduction, while sporozoite prevalence did not. Far fewer mosquitoes were examined for oocysts than sporozoites, and sampling variation thus contributes more to the oocyst data.

The increase in oocyst prevalence thus seems very unlikely to be related to the change in drug policy. Nor is it likely that any substantive change in coverage of bed nets could have contributed to the observed difference in oocyst rates because net ownership and use remained relatively low and stable in Rufiji District until late 2005. There were no major changes in availability of nets in Kilombero-Ulanga during the study period [30].

Although an efficacious ACT with known gametocytocidal properties was deployed and achieved reasonable population level coverage with an estimated 0.6 to 2.2 AS + SP treatments per person per year, the majority of persons receiving treatment with ACT were symptomatic children. Thus, the asymptomatic, chronically infected, semi-immune older children and adults who likely constituted the bulk of the reservoir of gametocytes [23] were relatively untouched by the introduction of ACT for case management. There have even been suggestions of higher infectivity of gametocytes in asymptomatic carriers in comparison to symptomatic cases due to the large quantity of gametocytes in the former group [42]. In areas where the initial level of malaria transmission is relatively low, the ratio of symptomatic to asymptomatic infections is higher, and larger proportionate reductions in transmission may be likely following introduction of ACT [6,39,43]. Conversely, in areas of high transmission such as investigated here, ACT may have little impact on prevalence, human population infectiousness and consequent mosquito-to-human transmission because a greater proportion of infections are only mildly symptomatic. Furthermore, even in settings such as these where artemisinins are combined with complementary partner drugs, such as SP which have long-lasting prophylactic effects [19], ACT use may have little impact on overall transmission where it occurs at high intensities simply because individuals often become re-infected within weeks of treatment [1].

ACT might only have a substantial effect on the infectious reservoir if most of the infections are actually being treated with this drug class. The delivery of ACT through public sector outlets in Rufiji rose steadily from 2003 to 2005 with a total of 450,000 doses being deployed for distribution to all registered health facilities by that time [44], corresponding to a mean consumption rate of 2.22 doses per person per year. Adherence among recipients has been estimated at 75% [45], which implies that this drug was delivered reasonably effectively. The proportion of care-seeking visits made to the health facilities that were fever-related rose from 31.8% in 2001 to 54.7% in 2004 [46], perhaps due to improved community perceptions, availability and affordability. Recent calls for accurately targeting ACT only to those with patent parasitaemia [47] may, paradoxically, further undercut the potential for case management alone to contribute to transmission reduction in highly endemic settings.

While much emphasis has been placed upon the importance of the gametocytocidal properties of ACT, their most important contribution to lowering human population infectiousness is to terminate otherwise long-lasting infections with asexual stages, which intermittently but persistently generate gametocytes and can infect mosquitoes for over a year [19]. This is comparable to the effect of non-gametocytocidal blood schizonticides. Similarly, the impact of curative drugs upon onward transmission is probably primarily determined by the length of time successfully treated patients remain uninfected and consequently non-infectious, rather than whether that drug kills the relatively short-lived gametocytes already present at the time of administration. Therefore, while an effective cure may reduce human population infectiousness in an area with little transmission, in parts of Africa where it is common to become re-infected within weeks or even days, even regular treatment of symptomatic infections [48,49] will likely have only a modest effect upon the proportion of people’s lives spent infected and, therefore, on the mean infection prevalence as described [25].

## Conclusions

Whilst it is disappointing that no obvious reduction of human infectiousness was evident after introduction of ACT for malaria case management in this first large-area trial in a region of intense transmission, perhaps this is not entirely surprising. Both rapid re-infection and semi-immune, chronically infectious, asymptomatic carriers are common in such settings. The lack of any such secondary benefits in high transmission areas should not detract from the direct public health value of ACT as a means to treat uncomplicated malaria and prevent severe disease manifestations. As has already been outlined in both theory [19] and practice [8,11], effective chemotherapy with ACT has a vital role in reducing malaria morbidity and mortality. The contribution of chemotherapy to the control and elimination of transmission is likely to be most valuable in settings where transmission is either naturally low or where other approaches such as effective vector control have brought it down to more tractable levels.

There is a need for entomological surveys in parallel to clinical surveillance as a routine component of large-scale trials of anti-malarial drugs or vaccines, but variations in space and time in entomological data should not ignored. Malaria parasite prevalence in vector populations may serve as a useful indicator of the population-wide effect of deployment of interventions that may have only previously been evaluated in individual participants in clinical trials. There is also a need for more cost-effective technologies and procedures for sampling vector mosquito populations across large areas [50,51] to enable accurate and precise measurement of their infection prevalence.

Finally, although there was no demonstrable impact of introducing ACT free for routine case management without diagnostic confirmation, this should not discourage malaria control programmes and their development partners from rolling out interventions to enhance ACT coverage and improve targeting through existing diagnostic tests. Since the study was conceived, ACT and effective vector control through insecticide-treated bed nets have been scaled up broadly, coinciding with substantial reductions in malaria-related and all-cause child mortality in areas of highly endemic malaria transmission [52]. These findings suggest that untargeted ACT alone may have limited impact on transmission. Endemic countries and their development partners should continue to promote ACT and confirmed diagnosis, but may wish to reconsider their expectations of what effect this may have on malaria transmission. Scaling-up and sustaining effective case management along with proven vector control interventions remains the priority for these areas.

## Abbreviations

ACT, Artemisinin Combination Therapy; CDC, Centers for Disease Control; CSP, Circumsporozoite Protein; EIR, Entomological Inoculation Rate; ELISA, Enzyme-Linked Immunosorbent Assay; IMPACT, The Interdisciplinary Monitoring Project for Antimalarial Combination Therapy; MTIMBA, Malaria Transmission Intensity and Mortality Burden Across Africa; NIMR, National Institute of Medical Research; SP, Sulphadoxine Pyrimethamine.

## Competing interests

The authors declare that they have no competing interests. The findings and conclusions in this report are those of the authors and do not represent the official position of the Centers for Disease Control and Prevention.

## Authors’ contributions

SMKA, TAS, SPK conceived and designed the study. JK and AK led the field data collection. BJH, GFK, TAS, and SMKA analysed and interpreted the data. BJH, GFK, HMF, TAS, SMKA, CL, SPK and JDC drafted the manuscript. GFK, AT, JK and AK provided administrative, technical, and material support. All authors read and approved the final manuscript.
